# Population status and ecology of the *Salmo trutta* complex in an Italian river basin under multiple anthropogenic pressures

**DOI:** 10.1002/ece3.6457

**Published:** 2020-06-09

**Authors:** Antonella Carosi, Lucia Ghetti, Rosalba Padula, Massimo Lorenzoni

**Affiliations:** ^1^ Department of Chemistry, Biology, and Biotechnologies University of Perugia Perugia Italy; ^2^ Forest, Economics, and Mountain Territory Service Perugia Italy; ^3^ Centre for Climate Change and Biodiversity in Lakes and Wetlands of Arpa Umbria Perugia Italy

**Keywords:** climate change, distribution range, flow rates, Mediterranean basins, Mediterranean trout, trout ecology

## Abstract

Salmonids inhabiting Mediterranean rivers are of particular concern for biodiversity conservation, as they are threatened by various stressors, including habitat alterations, overfishing, climate change, and introgressive hybridization with alien species. In the Tiber River basin (Central Italy), genetic introgression phenomena of the native *Salmo cettii* with the non‐native *Salmo trutta* hinder the separate analysis of the two species, which are both included in the *S. trutta* complex. Little is known about the factors currently limiting the trout populations in this area, particularly with respect to climate change. With the intention of filling this gap, the aims of the current study were to (a) quantify changes in the climate and (b) analyze the distribution, status, and ecology of trout populations, in the context of changing abiotic conditions over the last decades. Fish stock assessments were carried out by electrofishing during three census periods (1998–2004, 2005–2011, and 2012–2018) at 129 sites. The trend over time of meteorological parameters provided evidence for increased air temperature and decreased rainfall. Multivariate analysis of trout densities and environmental data highlighted the close direct correlation of trout abundance with water quality, altitude, and current speed. Climate‐induced effects observed over time in the sites where trout were sampled have not yet led to local extinctions or distribution shifts, indicating a marked resilience of trout, probably due to the buffering effect of intrinsic population dynamics. Decreasing body conditions over time and unbalanced age structures support the hypothesis that variations in hydraulic regime and water temperature could overcome these compensatory effects, which may lead to a severe decline in trout populations in the near future. In a climate change context, habitat availability plays a key role in the distribution of cold‐water species, which often do not have the possibility to move upstream to reach their thermal optimum because of water scarcity in the upper river stretches.

## Graphical abstract

The survival of the native Mediterranean trout *Salmo cettii* is threatened by various anthropogenic stressors, including the genetic introgression with the alien *Salmo trutta* and the effects of climate change. The aims of the study were to quantify changes in the climate and to analyze distribution, status and ecology of *S. trutta* complex populations in the upper Tiber River basin (central Italy). The absence of significant range shifts for trout populations indicates a marked resilience of trout populations probably due to buffering effects of intrinsic population dynamics. However, strong variations in hydraulic regime and water temperature could overcome these compensatory effects and may lead to a severe decline in trout populations in the near future.

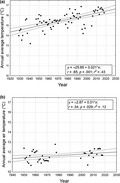

## 1 INTRODUCTION

In Europe, over 25 species belonging to the genus *Salmo* (Linnaeus, 1758) are reported (Kottelat, 1998; Tougard, Justy, Guinand, Douzery, & Berrebi, 2018), and all of them are of particular interest from a socio‐economic point of view. Salmonids include game species, which are particularly important in sport fishing activities worldwide, and play a key role in the economy of outdoor recreation (Brown, Lokensgard, Snyder, & Draper, 2019; Splendiani, Ruggeri, Giovannotti, & Caputo Barucchi, 2013). Because of their notable recreational and economic value, particular attention is paid to the conservation and management of fish in the genus *Salmo* (Filipe et al., 2013). In Italy, several trout genetic lineages belong to the genus *Salmo* (Lobón‐Cerviá et al., 2019). The native species derived from colonization processes which occurred in the past geological eras, mostly influenced by Pleistocene glaciations, while non‐native species are represented by domestic trout of Atlantic origin, introduced by restocking activities (Caputo, Giovannotti, Nisi Cerioni, Caniglia, & Splendiani, 2004; Meraner & Gandolfi, 2018; Splendiani et al., 2019).

**FIGURE 1:**
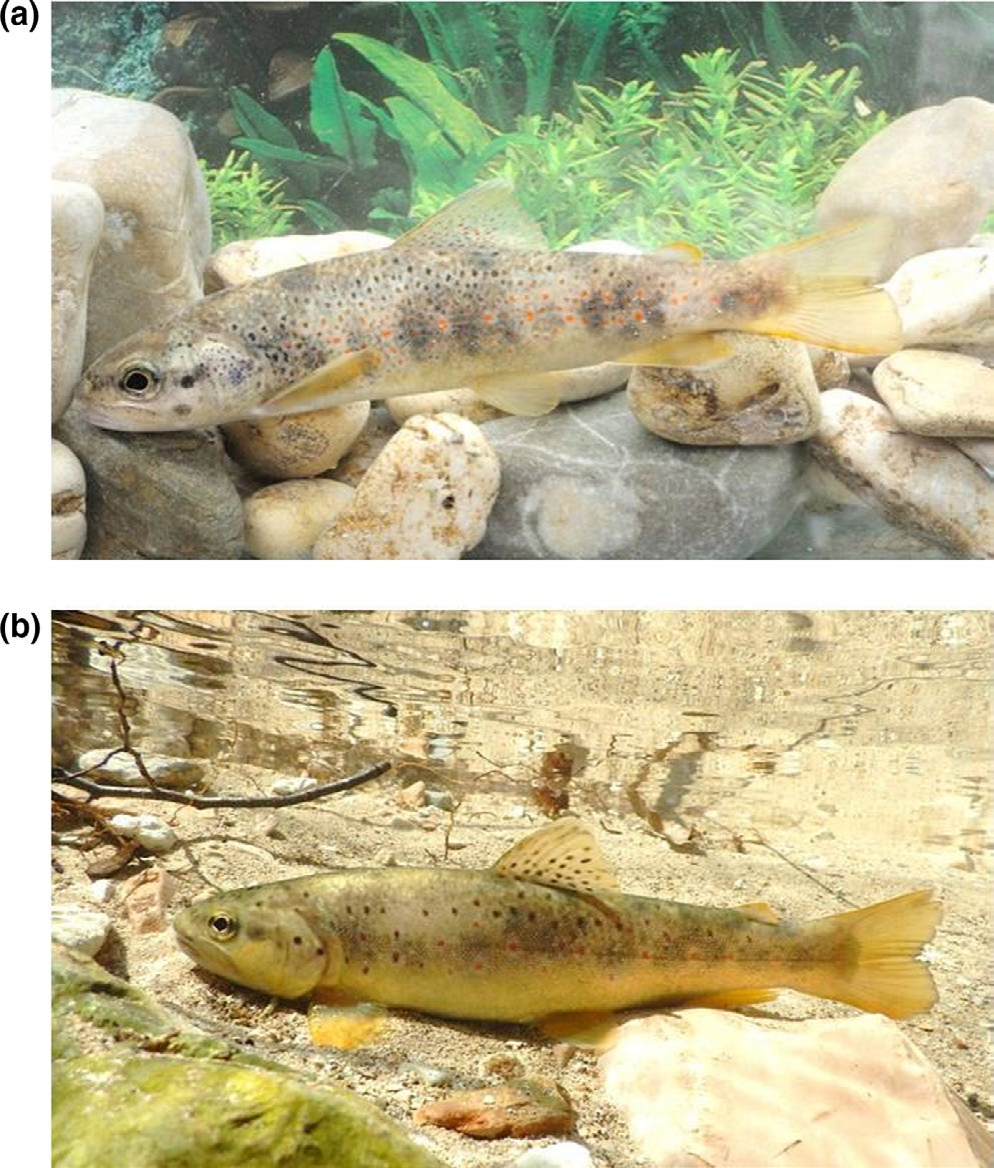
Photograph of (a) Mediterranean trout, *Salmo cettii* and (b) brown trout, *Salmo trutta*

In the Tiber River basin (Central Italy), there are two species belonging to the genus *Salmo*: the native Mediterranean trout, *Salmo cettii* Rafinesque, 1,810 (Figure 1a), and the non‐native Atlantic lineage brown trout, *S. trutta* Linnaeus, 1758 (Bernatchez, 2001; Patarnello, Bargelloni, Caldara, & Colombo, 1994) (Figure 1b). The Mediterranean trout is strongly compromised and currently reduced to a small number of populations due to introgressive hybridization with *S. trutta* (Splendiani et al., 2019). Splendiani et al. (2016) reported that <3% of Apennine trout populations in Central Italy are exempt from introgression phenomena. In the northern section of the Tiber River basin, only three residual Mediterranean trout populations are genetically pure, and they inhabit the Torsa, Monterivoso, and Vigi creeks in the mountainous part of the Nera River sub‐basin (Lorenzoni et al., 2019a; Lucentini et al., 2006; Mearelli et al., 1995; Splendiani et al., 2019). In the southern part of the Tiber River basin, the presence of only one native population in the Aniene River sub‐basin (Simbrivio Creek) was recently reported (Martinoli et al., 2019; Rossi et al., 2019). All the other populations are characterized by relatively high levels of introgressive hybridization with *S. trutta* (Splendiani et al., 2019). For these reasons, in the present paper, we refer to the *S. trutta* complex, which includes *S. cettii* and *S. trutta,* since it is not possible to analyze the two species separately. Little is known about the factors currently limiting this species complex, particularly with respect to climate change. Despite the uncertainty of hybridization, the analysis of the *S. trutta* complex can be a way to expand our knowledge of the ecology of trout populations, and to offer a path forward in describing their adaptive changes in the context of changing abiotic conditions over the last decades.

In fact, in addition to the introduction of *S. trutta*, the survival of native trout populations in the Tiber River basin is threatened by various other anthropogenic stressors acting simultaneously, including water pollution, water extraction, overfishing, and river fragmentation (Lorenzoni et al., 2019a). Furthermore, since trout are cold‐water fish, they are particularly vulnerable to the effects of global warming, including increasing water temperatures and decreasing flow rates (Vera, Garcia‐Marin, Martinez, Araguas, & Bouza, 2013). These effects may be particularly severe in Apennine watercourses of modest dimensions in terms of width, depth, and flow rates (Lorenzoni, Barocco, Carosi, Giannetto, & Pompei, 2014). Furthermore, water scarcity conditions are expected in the future in the Mediterranean area and should be taken into consideration (Schewe et al., 2014).

Recent studies conducted in Europe have documented the effects of multiple anthropogenic pressures on trout, in terms of population declines and distribution shifts (Almodovar, Nicola, Ayllόn, & Elvira, 2012; Filipe et al., 2013; Hansen, Fraser, Meier, & Mensberg, 2009). Some studies, focusing on wild trout populations of the Mediterranean area, highlighted the need to take concrete actions to preserve the few residual native gene pools (Araguas et al., 2008, 2017; Vera et al., 2013). However, despite the remarkable conservation interest of the residual Mediterranean trout populations in Central Italy, their status and ecology are still not well documented. In addition to the genetic characterization of populations, it is important to expand our knowledge of changes that have occurred over time in their distribution, demography, and in their habitat characteristics, in order to properly manage the rivers inhabited by salmonids, not only at the local scale, but also in all of the Mediterranean area. Therefore, with the intention to fill this gap, the aims of the present study were to (a) quantify changes in the climate in areas with Apennine watercourses over the last decades and (b) analyze the distribution, status, and ecology of the *S. trutta* complex populations in the upper Tiber River basin, using a large data set, and describe their adaptive changes in the context of ongoing climate warming.

## 2 MATERIAL AND METHODS

### 2.1 Study area

**FIGURE 2:**
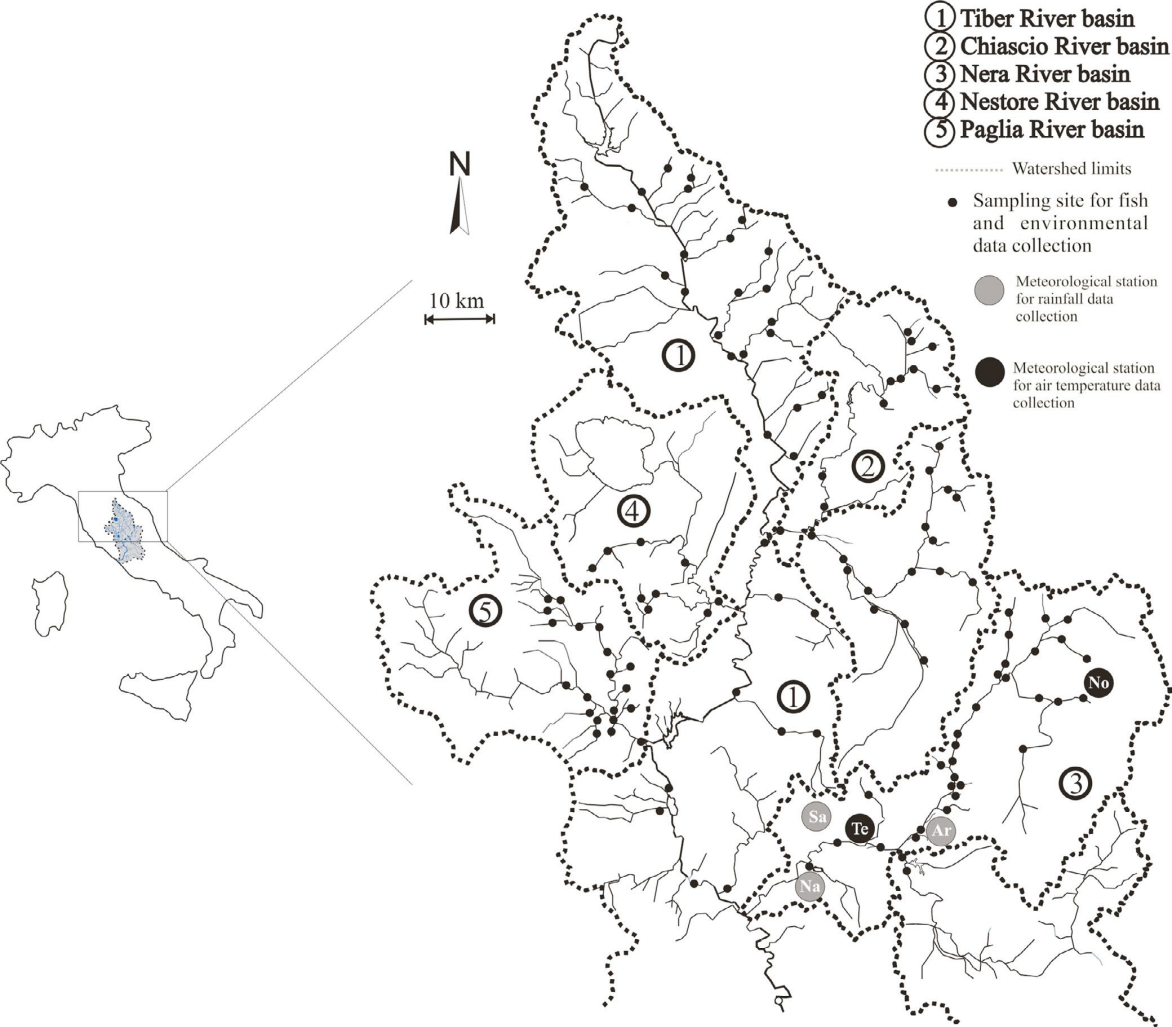
Study area and location of sampling sites

The study area comprised 92 watercourses of the upper Tiber River basin, the second‐largest watershed in Italy. The whole area, which covers 8,412 km^2^, has been divided into five sub‐basins, namely the Chiascio, Nera, Nestore, Paglia, and Tiber River basins (Figure 2). These sub‐basins have different characteristics in terms of soil permeability and water quality (Carosi, Ghetti, La Porta, & Lorenzoni, 2017). The upper section of the Tiber River basin is characterized by a mean permeable surface (i.e., surface area developed on tufa and calcareous bedrock) equal to 53%, and an average altitude of 742 m a. s. l.. The Nestore and Paglia River basins are characterized by a lower permeability (33%) and their watercourses flow in hilly areas (average altitude: 389 m a.s.l.). All of these streams have poor water quality and torrential characteristics, with marked flow rate oscillations and drought periods in summer, which are exacerbated by water extractions for irrigation purposes (Carosi, Ghetti, Forconi, & Lorenzoni, 2015; Carosi, Padula, Ghetti, & Lorenzoni, 2019). The Nera River basin is mountainous (average altitude: 909 m a.s.l.) and permeable (85%). Its hydrographic network is composed of few watercourses with relatively stable flow rates throughout the year, thanks to the water supply guaranteed by many underground springs. The Chiascio River basin shows intermediate geomorphological features (average altitude: 909 m a.s.l.; mean permeable surface: 59%). The whole study area is characterized by a high degree of river fragmentation due to the presence of 188 weirs and seven dams spread throughout the hydrographic network (Carosi, Padula, et al., 2019). Recent studies (Carosi, Ghetti, Padula, & Lorenzoni, 2019; Carosi, Padula, et al., 2019) provided some evidence that changes in the local climate have induced direct effects on the water bodies in the study area, in terms of increasing water temperatures and decreasing the annual average flow rates. Recreational trout fishing is widespread in the area, mainly in the Nera and Chiascio River basins.

### 2.2 Data collection

To address our first objective, daily cumulative rainfall data were collected from 1920 to 2018 at three meteorological stations located at Arrone, Narni Scalo, and Sangemini villages (coded as Ar, Na, and Sa, respectively, in Figure 2). Mean daily air temperature data were collected from two meteorological stations, located in Terni city (data collection from 1929 to 2018) and Norcia city (from 1950 to 2018), coded as Te and No, respectively (Figure 2). Rainfall and air temperature data were provided by the Hydrographic Service of the Umbria Region and by the Department for the Protection of Inland and Marine Waters of the Italian Institute for Environmental Protection and Research. To have data available for a time period exceeding that of the fish censuses, and to offer a more significant trend, air temperature was used as a surrogate for water temperature and as a proxy for potential evapotranspiration. Given the strong link between precipitation and flow rates in the study area, rainfall was used as a proxy for water discharge in the rivers. The meteorological stations were chosen based on the length of the historical data series available. Air temperature calculations were performed using average monthly data, while for rainfall, annual cumulative data were used.

To address our second aim, we assembled fish and environmental data collected during the census periods 1998–2004, 2005–2011, and 2012–2018, at 129 sampling sites. As the environmental changes along the longitudinal gradient of the rivers are often associated with changes in the composition of fish assemblages (Huet, 1954; Vannote, Minshall, Cummins, Sedell, & Cushing, 1980), the collection sites were selected to represent river stretches characterized by the same slope. A census of the fish fauna was carried out by electrofishing at each sampling location, using the two‐pass removal method (Zippin, 1956). The length of sample sites was set as 10 times the mean width (Bayley & Peterson, 2001). The surveyed areas varied from a minimum of 40 m^2^ to a maximum of 2,046 m^2^ (mean ± *SE* = 571.75 ± 16.57). Each site was sampled three times, one for each census period, and sampling was conducted in the autumn, during the morning. The sites were sampled in the same sequence within each census period, so the gap between the three sampling occasions was 7 years for each site. For each sampled specimen, total length (TL, ±0.1 cm) and weight (±0.1 g) were measured. The identification of the species was based on the results of previous genetic characterization projects carried out in the study area (Lorenzoni et al., 2019a; Lucentini et al., 2006; Mearelli et al., 1995; Splendiani et al., 2019). A sample of scales was collected from each trout for age determination. Approximately 10–15 scales were taken posterior to the dorsal fin and above the lateral line (DeVries & Frie, 1996), on the left side of the body. When there were large numbers of sampled specimens, the scales were collected from a subsample for each increase in length of 1 cm to cover all length classes in the trout population. At the end of the field activities, all captured fish were released into their natural environment. The scales were stored in ethanol (30%) for later observation under a stereomicroscope using the image‐analysis system IAS 2000. For each trout, age was determined independently by two observers, using the scalimetric method (Bagenal, 1978), and further validated by means of length–frequency distribution (Britton, Cowx, & Peirson, 2004). For each population, the density (ind/ha) and standing crop (g/ha) were estimated. The population density was calculated by dividing the estimated number of fish belonging to each age group by the surface area of the sampling site.

To characterize the river stretches, 18 environmental parameters were measured at the same time as the fish samplings, including data on the macroinvertebrate community composition, for which the extended biotic index (EBI) was applied (Ghetti, 1986) (for more details about data sampling and laboratory analyses, see Carosi, Ghetti, & Lorenzoni, 2016). Conductivity (μS/cm), pH (units), water temperature (°C), and dissolved oxygen (mg/L) were measured in the field at the same time as fish samplings, using electronic meters manufactured by YSI, Hanna Instruments, and WTW GmbH, respectively. The hydrological parameters (flow rate (m^3^/s) and current speed (m/s) were measured at the cross‐sectional area of each sampling reach, using a current meter. The average current speed was calculated by dividing the flow rate value by the cross‐sectional area (m^2^). Other chemical parameters of the water (chlorides, sulfates, phosphates, and ammonia) were subsequently measured in the laboratory. Watershed area (km^2^), distance from the water source (km), and altitude (m a.s.l.) were derived from digital maps using GIS (geographic information system) provided by the Forest, Economics, and Mountain Territory Service of the Umbria Region.

### 2.3 Trout body condition and population size structure estimation

We assessed the relative weight (*W*_r_) to estimate changes in body condition of trout in the *S. trutta* complex over time. *W*_r_ was calculated using the following equation: *W*_r_ = 100 × (*W*/*W*_s_), where *W* = body mass, and *W*_s_ = standard weight (i.e., the length‐specific ideal biomass predicted by a length–mass regression calculated for a whole species to represent populations in better‐than‐average physiological conditions, Murphy, Willis, & Springer, 1991). The relative weight (*W*_r_) is a condition index based on the comparison between the real weight of an individual and the optimal weight (*W*_s_). *W*_r_ values lower than 95 indicate poor body condition (Blackwell, Brown, & Willis, 2000; Murphy, Brown, & Springer, 1990). *W*_r_ estimation allows evaluation of the physiological status of fish (Brown & Murphy, 1991), to compare specimens or populations of different lengths, and to highlight the occurrence of ecological changes over time (Blackwell et al., 2000). In the present study, the standard weight *W*_s_ was estimated using the following equation calculated for the *S. trutta* complex in the Tiber River basin using the empirical percentile (EmP) method, which is not influenced by the size of the specimens (Angeli et al., 2010; Gerow, 2010), as follows:

$$\log_{10}\left(W_{s}\right)=-5.203+\left(3.154\log_{10}\text{TL}\right)-\left(0.015{\left(\log_{10}\text{TL}\right)}^{2}\right);$$

where the TL application range (cm) is 8–58.

We assessed the proportional stock density (PSD) index (Gabelhouse, 1984) to provide a numeric estimation for deviations of the *S. trutta* complex population structure from a balanced population. We calculated PSD using the following equation: PSD = 100 × (number of fish ≥ minimum quality length/number of fish ≥ minimum stock length). The minimum quality length was defined as the minimum size of fish that most recreational fishermen prefer to catch, while the minimum stock length was defined as the approximate length at sexual maturity (Anderson & Neumann, 1996). In this study, to establish the minimum quality length (TL = 25 cm) and the minimum stock length (TL = 22 cm), the values indicated by Pedicillo, Carosi, Ghetti, and Lorenzoni (2010) for the trout populations of Central Italy were used. The PSD values varied from 0 to 100, and the optimal range for a balanced population is 35 ≤ PSD ≤65 (Gabelhouse, 1984; Gassner, Tischler, & Wanzenbock, 2003).

### 2.4 Data analysis

#### 2.4.1 Changes in climate

In order to evaluate temporal changes in precipitation and air temperature (dependent variables), linear regressions with years (independent variable) were performed. The significance of each relationship was tested using analysis of variance (ANOVA), and the significance of the regression coefficient was tested using *t* test analysis.

To highlight changes in the mean water temperature and average current speed in the sites in which trout were sampled over the three census periods (1998–2004, 2005–2011, and 2012–2018), ANOVA was performed.

#### 2.4.2 Distribution of trout populations

To analyze the relationships among environmental parameters and trout density in the investigated area, principal component analysis (PCA) was performed. The data matrix included 20 variables (basin‐based soil permeability, altitude, distance from the source, watershed area, flow rate, average current speed, water temperature, conductivity, pH, dissolved oxygen, biochemical oxygen demand (BOD_5_), nitrate (NO_3_‐), nitrogen dioxide (NO_2_), ammonia (NH_3_), phosphates (PO_4_^3−^), sulfates (SO_4_^2−^), chlorides (Cl^−^), fragmentation degree, EBI, and *S. trutta* complex density) and 387 observations (129 sites × three sampling events). The degree of river fragmentation has been codified as the number of weirs with height > 80 cm present downstream until confluence with the mainstream river. Soil permeability was determined from the Italian geo‐lithological map at a scale of 1:500,000 (WMS Service − pcn. Minambiente.it). All the variables (*N*) were transformed (log_10_(*N* + 1)) to normalize their distribution (Brown & Austen, 1996).

In order to display possible changes in the distribution of trout populations, a map of the trout distribution and abundances related to the 1998–2004, 2005–2011, and 2012–2018 census periods is provided. An occupancy–elevation plot was constructed using trout presence data at different altitudes.

#### 2.4.3 Detectability, occupancy, colonization, and local extinction probabilities

Following Eby, Helmy, Holsinger, and Young (2014), on the basis of *S. trutta* complex presence/absence data, a multi‐sampling period model was used to estimate detectability (*p_it_* = probability that the species will be detected at site *i* at time *t*, given presence), occupancy (*ψ_i_* = probability that the species is present at site *i*) (MacKenzie et al., 2002), colonization (*ɣ*), and local extinction (*ε*) probabilities in the study area. The estimation was performed using presence–absence data for 129 survey sites across three sampling periods (period 1:1998–2004; period 2:2005–2011; and period 3:2012–2018). Changes in occupancy over time were evaluated. Elevation and river fragmentation were used as site covariates. Since both average current speed and water temperature (point estimated) change over time, they were used as sample covariates. Akaike's information criterion (AIC, Burnham & Anderson, 1998) was used to select the best candidate model. Following Burnham and Anderson (2003), all models within two AIC units were considered as a set of parsimonious models. Covariates for which 95% confidence intervals (*α* = .05) overlapped zero and with β/SE absolute values < 1.4 were considered uninformative (Arnold, 2010). The analysis was performed using the PRESENCE program for Windows.

#### 2.4.4 *S. trutta* complex population density, body condition, and size structure changes

Since for the *S. trutta* complex population density, young‐of‐the‐year (YOY) density, *W*_r_, and PSD were estimated for the same sites across three periods (1998–2004, 2005–2011, and 2012–2018), in order to test their changes over time, a one‐way repeated measures ANOVA was performed. Data were tested for sphericity using the Mauchly test, and the Greenhouse–Geisser and Huynh–Feldt adjustments were applied. All the statistical analyses mentioned above were performed using Dell STATISTICA 13 software for Windows.

## 3 RESULTS

**FIGURE 3:**
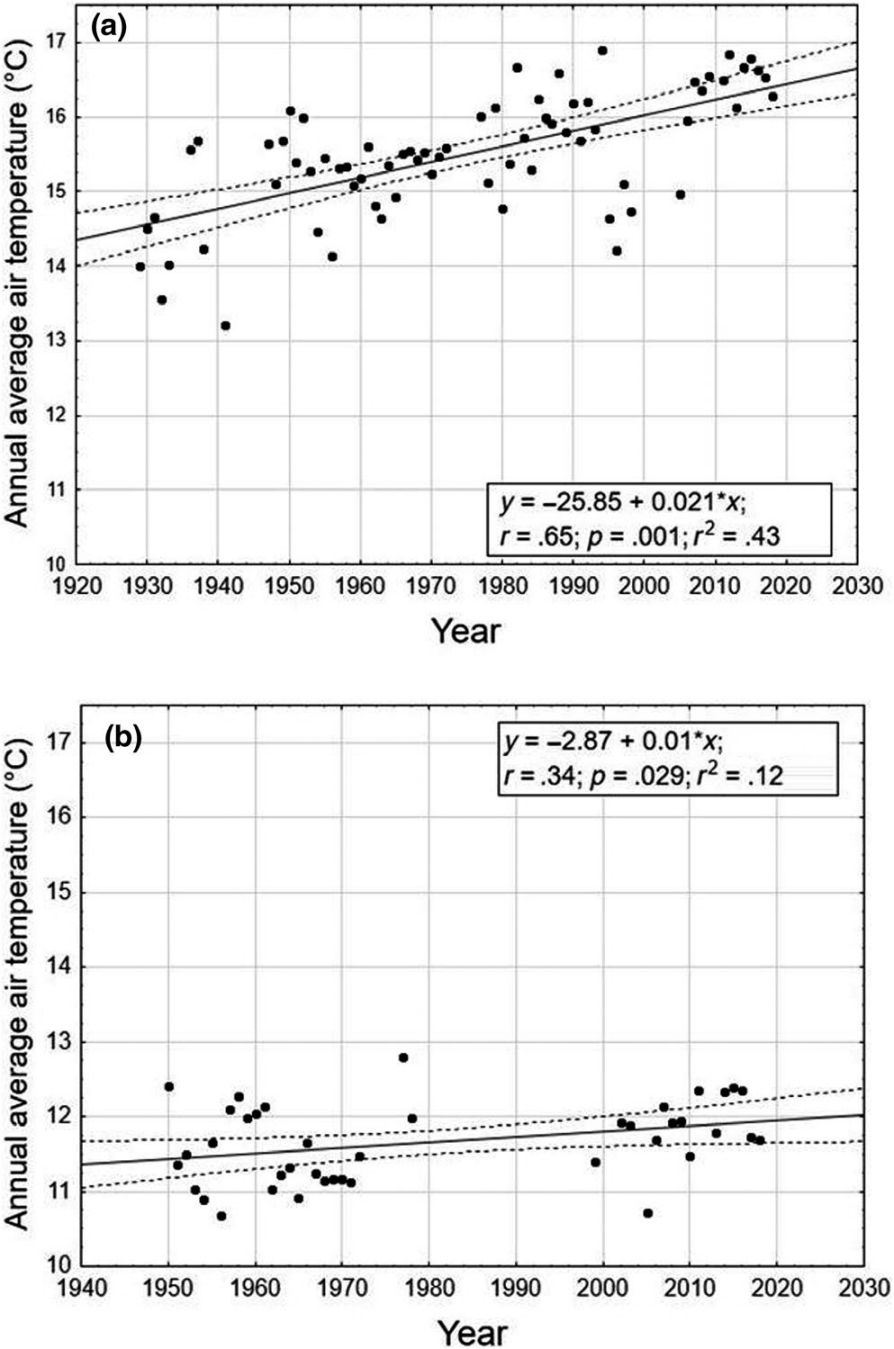
Trend over time of air temperature for automatic meteorological stations located at (a) Terni (130 m a.s.l., temperature measured from 1929 to 2018) and (b) Norcia (604 m a.s.l., temperature measured from 1950 to 2018). Dashed lines represent the regression bands with 0.95 confidence intervals (*α* = .05)

**FIGURE 4:**
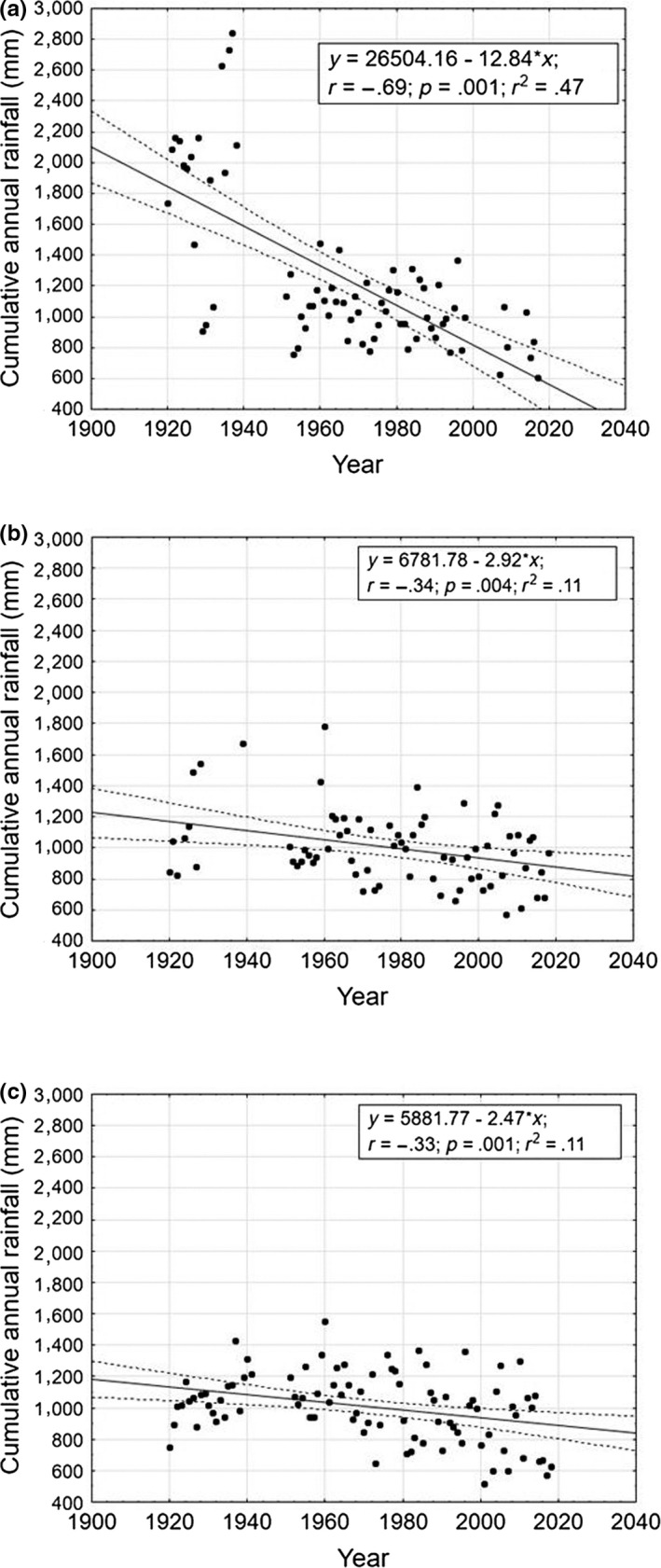
Trend over time of annual average rainfall for (a) Arrone (243 m a.s.l.), (b) Narni Scalo (240 m a.s.l.), and (c) Sangemini (337 m a.s.l.). Time period: years 1920–2018. Dashed lines represent the regression bands with 0.95 confidence intervals (*α* = .05)

The air temperature values (years 1951–2016) showed an increasing trend over time for both meteorological stations (Figure 3). The linear regressions were significant both using the *t* test analysis (Terni: *t* = 7.1, *p* = .001; Norcia: *t* = 2.66; *p* = .029) and using ANOVA (Terni: *F* = 50.28, *p* = .001; Norcia: *F* = 5.13, *p* = .029). The annual cumulative rainfall data showed a highly statistically significant decreasing trend over time in all the sites using the *t* test analysis (Arrone: *t* = 7.95, *p* = .001; Narni Scalo: *t* = 2.99, *p* = .004; Sangemini: *t* = 3.31, *p* = .001) and ANOVA (Arrone: *F* = 63.16, *p* = .001; Narni Scalo: *F* = 3.95, *p* = .004; Sangemini: *F* = 10.93, *p* = .001). This effect was particularly marked for the Arrone meteorological station (Figure 4).

**FIGURE 5:**
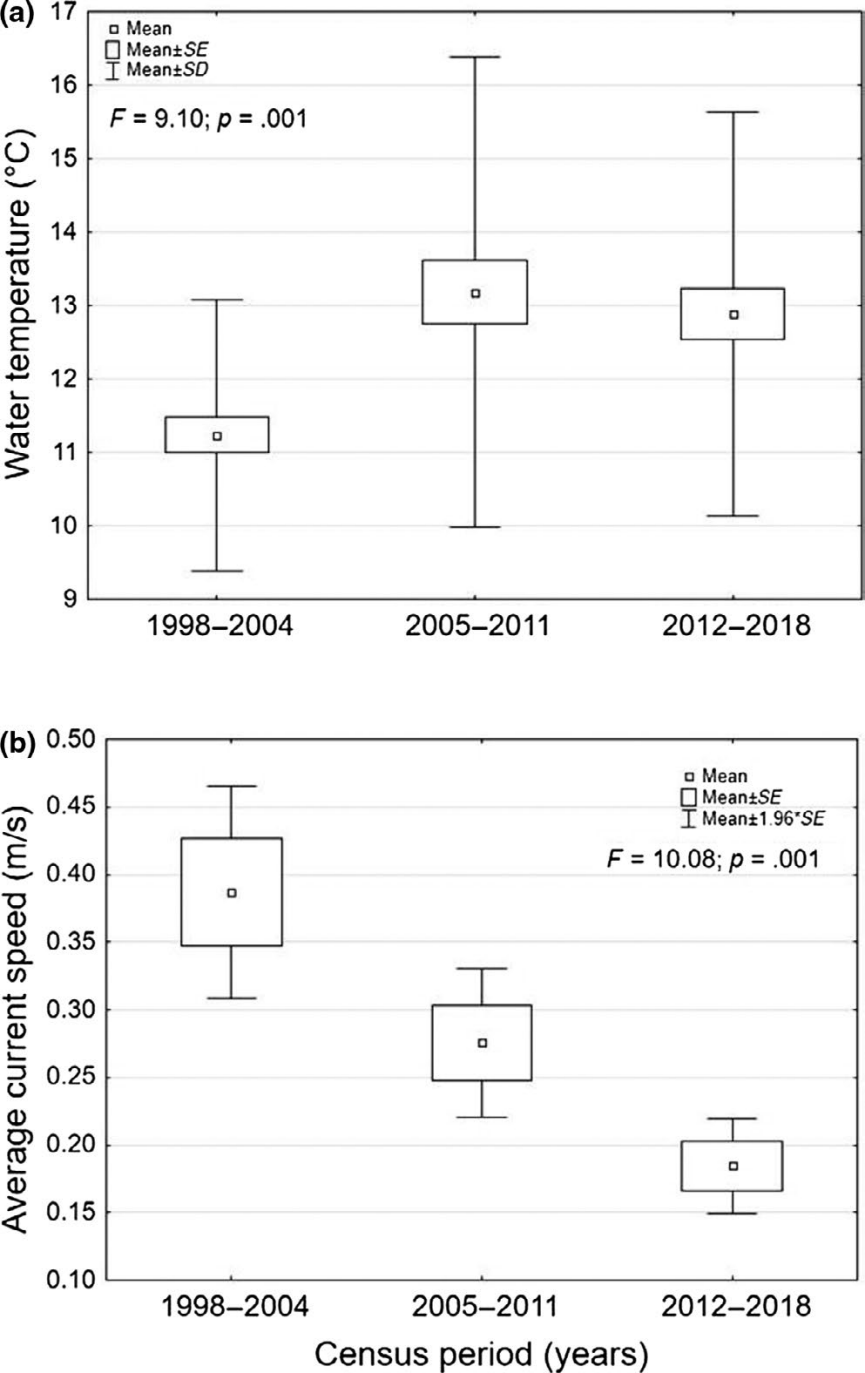
Trend over time for (a) water temperature, (b) average current speed, considering only the sites in which trout were sampled over the census periods 1998–2004, 2005–2011, and 2012–2018

Over the three census periods (1998–2004, 2005–2011, and 2012–2018), the comparison among the mean water temperature values of sites in which the trout species were present, carried out by ANOVA, showed a highly statistically significant increasing trend in more recent years than in the past (Figure 5a). In addition, the comparison of average current speed values detected in the different periods in the sites in which the trout were sampled revealed a marked, highly significant decreasing trend over time (Figure 5b).

A total of 16,542 specimens in the *S. trutta* complex were collected, including 3,304 from the Chiascio River basin, 12,177 from the Nera River basin, 16 from the Nestore River basin, 203 from the Paglia River basin, and 842 from the Tiber River Basin. The size of the sampled fish ranged from 3.0 to 58.0 cm (mean ± *SE* = 14.50 ± 0.05) and the weight ranged from 0.3 to 2,335.0 g (mean ± *SE* = 60.63 ± 0.07). Eleven age classes (0+ to 10+ years) were identified. In all census periods, the most represented age class was the YOY age class (Appendix S1), which always constituted over 50% of the total sample, with the highest density value (ind/ha) detected in more recent years (mean ± *SE* = 4,761 ± 2,693).

**FIGURE 6:**
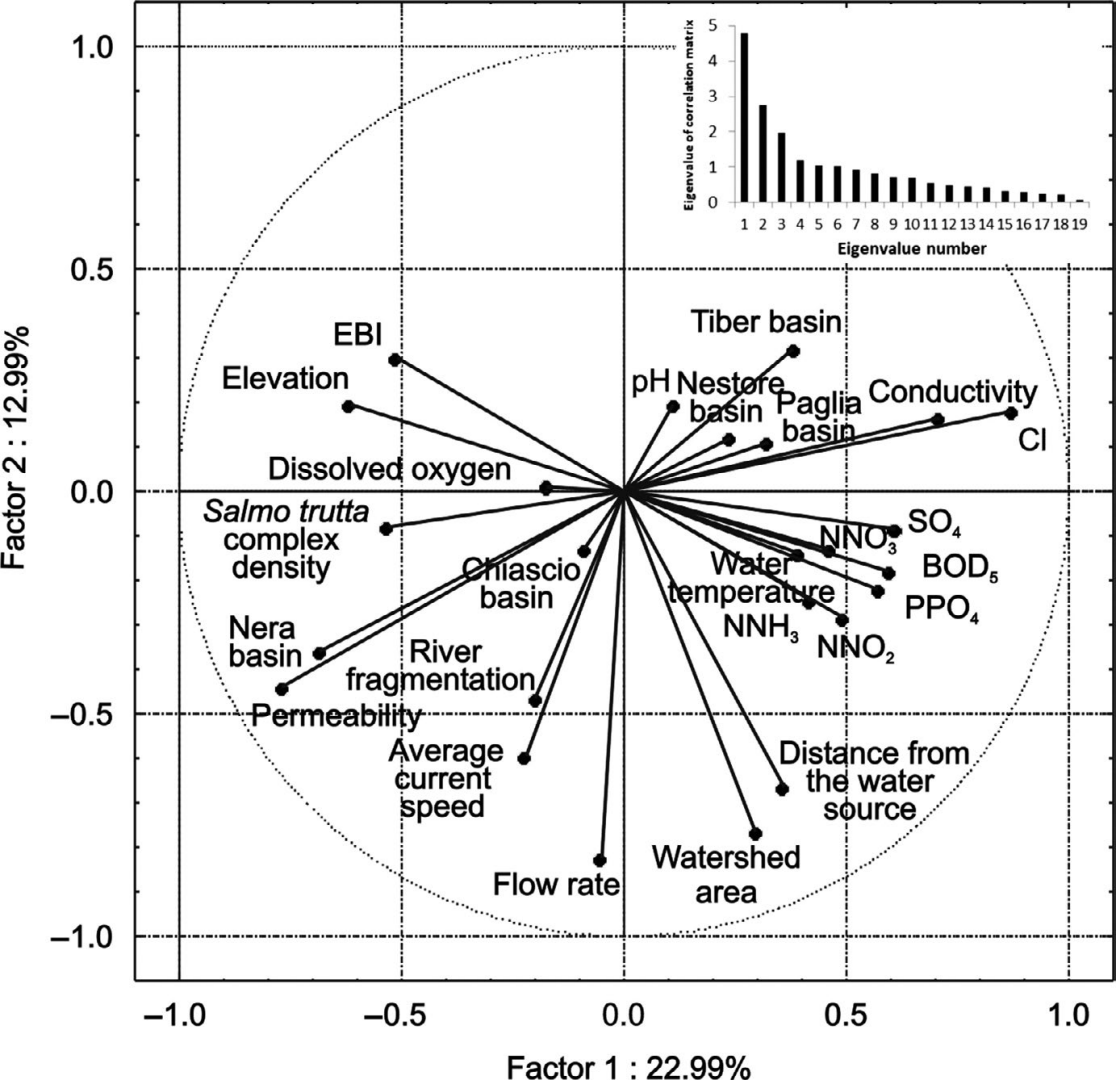
Principal component analysis (PCA) results: projection of the variables on the factorial plane factor 1 × factor 2

The first component of the PCA explained 22.99% of the overall variability and showed a strong positive correlation with distance from the source, watershed area, and all chemical–physical parameters, as well as a negative relationship with EBI, elevation, dissolved oxygen, and trout population abundance (Figure 6). This axis is representative of the upstream–downstream gradient of the river, where decreasing altitude corresponded to an increase in salt content, and a reduction in water quality. The Nera River basin and, to a lesser extent, the Chiascio River basin, were positively related to soil permeability. The second component was less informative (12.99% of the overall variability) and showed negative correlations with the average current speed and flow rate, and with conductivity and chlorides. This axis seems to reflect the increase in dilution capacity of the watercourses caused by increases in the amount of water.

**FIGURE 7:**
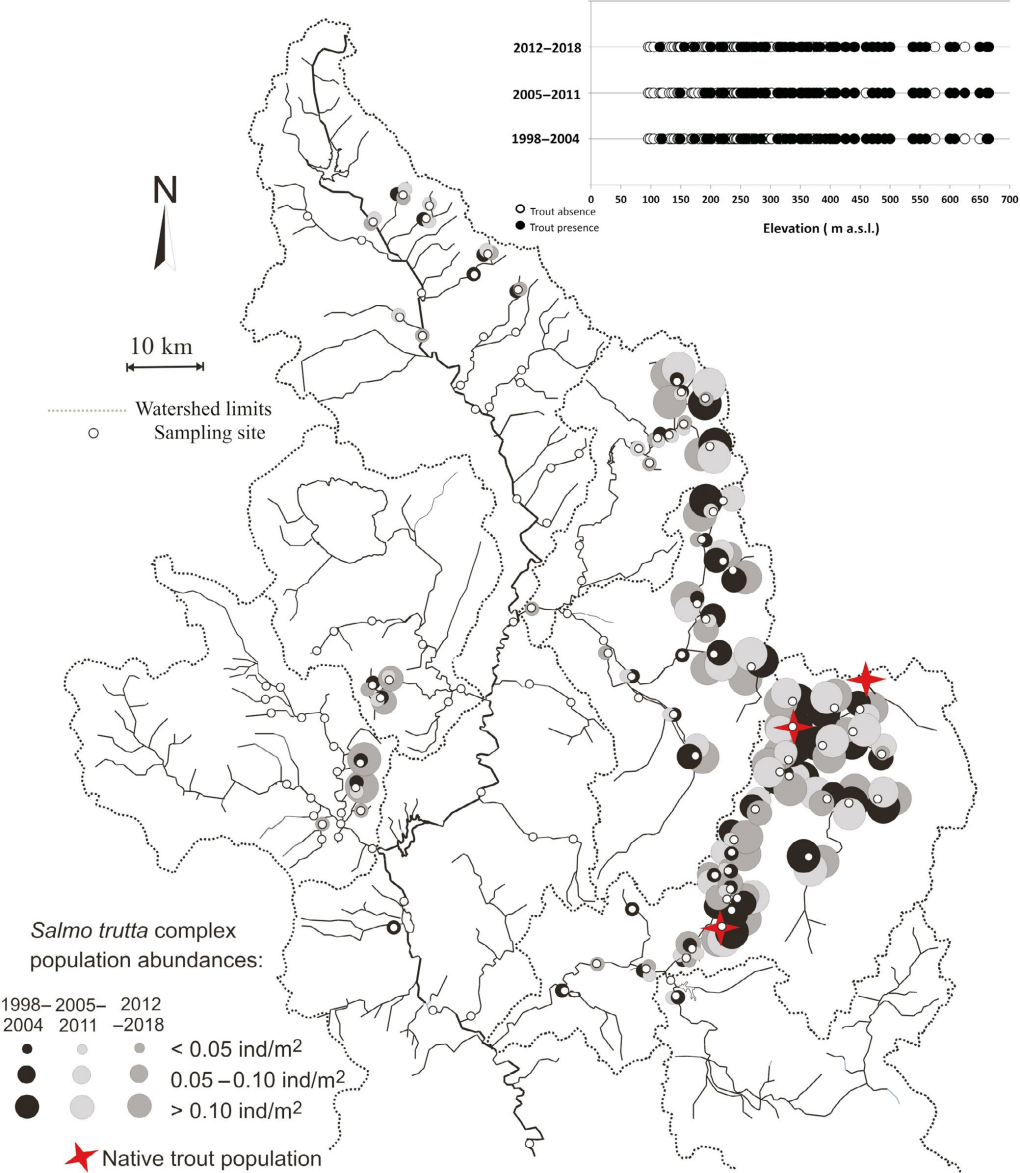
*Salmo trutta* complex distribution and occupancy–elevation plot

The comparison between trout distribution maps related to the three census periods showed no substantial changes over time, in terms of presence and abundance of *S. trutta* complex populations in the study area (Figure 7). The most abundant trout populations inhabit the mountainous part of the eastern tributaries of the Tiber River. The number of sites in which the *S. trutta* complex was present remained almost unchanged over time (60 sites from 1998 to 2004, 58 sites from 2005 to 2011, and 61 sites from 2012 to 2018).

**FIGURE 8:**
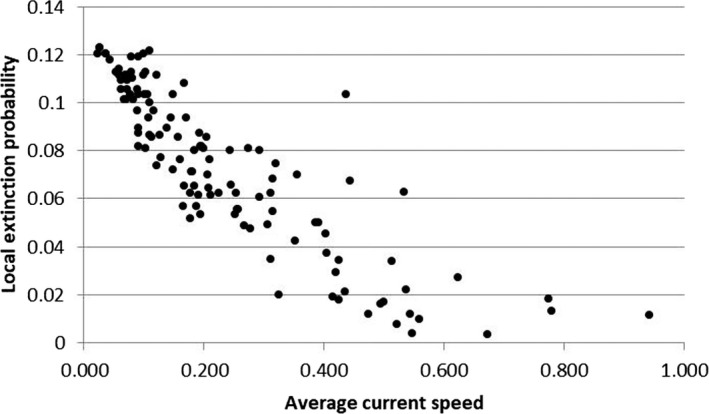
Effects of average current speed on local extinction probabilities for the *Salmo trutta* complex

**FIGURE 9:**
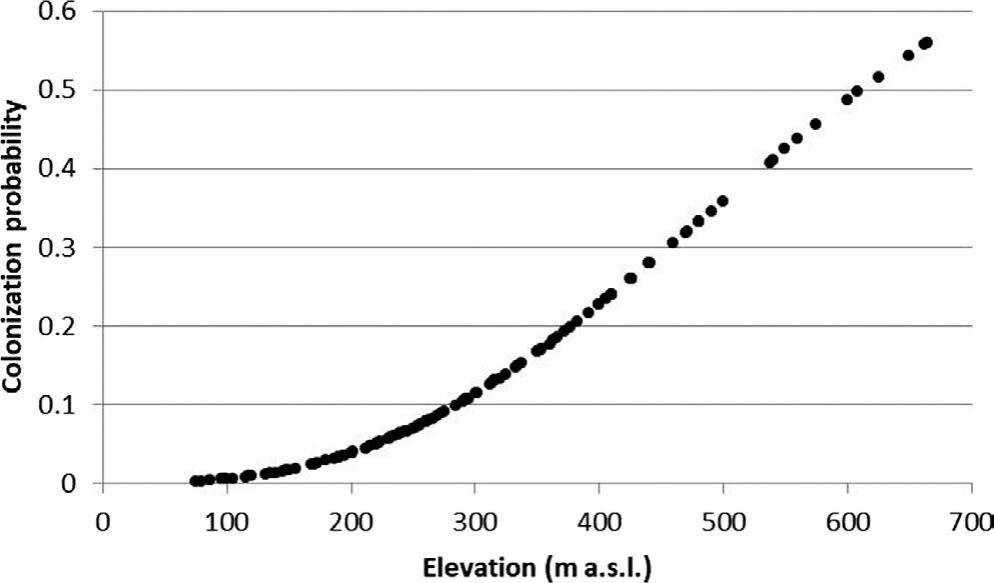
Effects of elevation on colonization probabilities for *Salmo trutta* complex

The detection probabilities for the *S. trutta* complex were quite high, ranging from 0.81 (years 1998–2004) to 0.86 (years 2012–2018). The occupancy probability remained almost unchanged over time, varying from 0.55 ± 0.057 to 0.57 ± 0.006, and the occupancy–elevation plot did not reveal trout range shifts toward river stretches located at higher altitudes (Figure 7). Both colonization and local extinction probabilities were quite low and equal to 0.63 ± 0.082 and 0.059 ± 0.038, respectively. The best multi‐sampling period models, within two AIC units of the top model, for estimating occupancy, colonization, local extinction, and detection, included elevation and average current speed as covariates (Table 1). The extinction probability (0.260 ± 0.026) decreased with the current speed, while colonization probability (0.07 ± 0.001) increased with elevation (Figures 8 and 9).

**TABLE 1:** Models within two Akaike information criterion (AIC) units of the top model for estimating occupancy (*ψ*), colonization (*ɣ*), local extinction (*ε*), and detection (*p*) probabilities for the *Salmo trutta* complex

Model	AIC	Delta AIC	AIC weight	Model Likelihood	n.Par.	−2 × LogLike
*ψ*, *ɣ* (year, elevation), *ε* (), *p* ()	400.81	0.00	0.18	1.00	7	386.81
*ψ*, *ɣ* (), *ε* (), *p* (year)	401.31	0.50	0.14	0.78	6	389.31
*ψ*, *ɣ* (),*ε* ( year), *p* ()	401.79	0.98	0.11	0.61	6	389.79
*ψ* (year), *ɣ* (),*ε* (), *p* ()	401.79	0.98	0.11	0.61	6	389.79
*ψ*, *ɣ* (year), *ε* (), *p* ()	401.79	0.98	0.11	0.61	6	389.79
*ψ*,* ɣ* (), *ε* (current speed), *p* ()	402.45	1.64	0.08	0.44	6	388.45

**FIGURE 10:**
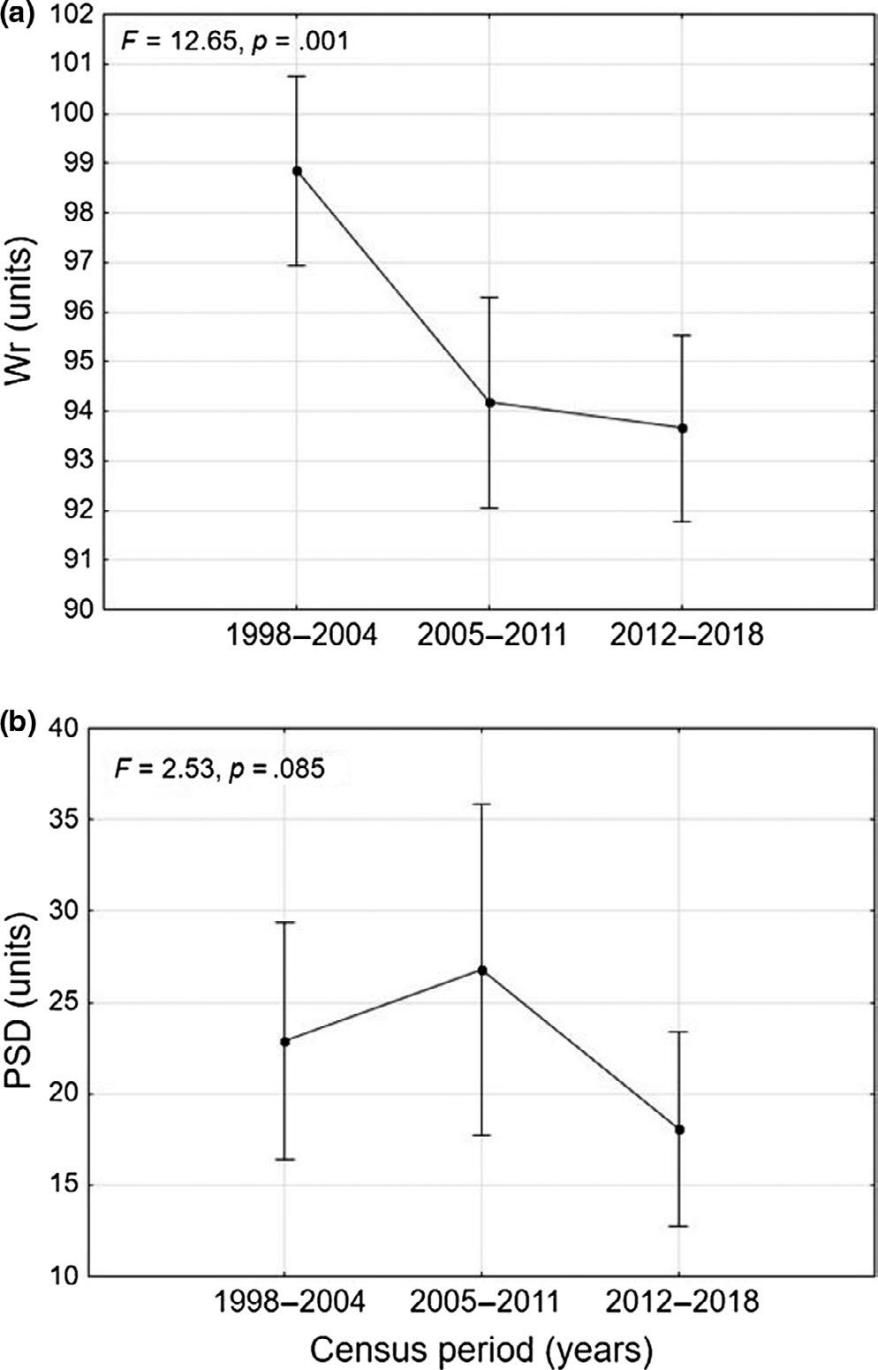
Trend over time of (a) relative weight and (b) proportional stock density (PSD) for the *Salmo trutta* complex over the census periods 1998–2004, 2005–2011, and 2012–2018

The repeated measures ANOVA, performed to evaluate the differences in mean density and standing crop values over time for the *S. trutta* complex, did not find statistically significant patterns (density: *F* = 1.97; *p* = .140; standing crop: *F* = 1.18; *p* = .310). In addition, the repeated measures ANOVA did not detect statistically significant differences between mean YOY densities (*F* = 1.49; *p* = .230). Regarding body condition, the repeated measures ANOVA showed a significant progressive decrease in *W*_r_ over the three census periods (*F* = 12.65, *p* = .001), with mean values remaining below the optimal range of 95–105 during the last two sampling periods (Figure 10a).

The PSD values were consistently below the optimal range (35–65), indicating imbalances in the population structures, with high prevalences of juveniles in the total sample (Figure 10b). However, the differences among the three census periods were not statistically significant (repeated measures ANOVA, *F* = 2.53, *p* = .085).

## 4 DISCUSSION

The trend over time in meteorological parameters provided evidence for some effects of global climate change, in terms of increased air temperatures and decreased rainfall, in the Nera River basin, where trout are more widespread. A recent study carried out in the Tiber River basin has already reported direct effects of climate change on watercourses flowing in the lowland stream reaches, in terms of increasing water temperatures and decreasing flow rates (Carosi, Padula, et al., 2019). These changes are already having effects on some parameters that greatly affect trout ecology, such as water temperature, flow rate, and current speed. In particular, in the sites where trout occur, a significant increase in water temperature over time and a drastic progressive decrease in current speed, exacerbated by the increasing water extractions, were observed. Altered hydrological regimes and stream temperature warming are known to play a key role in freshwater species distributional changes under climate change conditions (Eby et al., 2014; Wenger et al., 2011). Decreasing flow rates could result in a decrease in available trout habitat and food availability, affecting demography and growth of trout populations (Ayllón et al., 2019; Comte & Grenouillet, 2013; Naman, Rosenfeld, & Richardson, 2016), while current speed plays a crucial role, especially in relation to population recruitment (Unfer, Hauer, & Lautsch, 2011). Cold‐water species such as trout are particularly vulnerable to rising water temperatures because their demography, growth, and persistence are closely related to thermal conditions (Crozier & Hutchings, 2014; Isaak & Rieman, 2013; Kovach et al., 2016). Moreover, in the evaluation of climate change effects on trout populations in the study area, it is necessary to consider the modest dimensions of the analyzed water courses: the Apennine streams, which are characterized by low depths and modest flow rates, are particularly affected by low precipitation and increases in temperature (Lorenzoni et al., 2014). In these cases, a decline in flow rates could lead to a steep decline in current speed (Rosenfeld, 2017).

The multivariate analysis of population densities and environmental data highlighted the close direct correlation of trout with water quality, altitude, and current speed. Trout in the *S. trutta* complex are mainly located in the hydrographic eastern basins of the Tiber River (i.e., the Nera and Chiascio River basins), where the species often gives rise to mono‐specific communities (Carosi et al., 2015; Lorenzoni, Mearelli, & Ghetti, 2006), and the environmental characteristics, which include cold‐water and stable hydrologic conditions, are suitable for salmonids, owing to their high soil permeability and the occurrence of underground springs in this area (Lorenzoni et al., 2019a, 2019b; Splendiani et al., 2013). The trout inhabit watercourses located in mountainous and sparsely populated areas, which guarantee good water quality.

Contrary to what has been observed for many species under climate warming conditions (Bellard, Bertelsmeier, Leadley, Thuiller, & Courchamp, 2012), and also for some cyprinid species in the Tiber River basin (Carosi, Padula, et al., 2019), no significant changes were found in the distribution ranges of trout, in terms of upstream distribution shifts. This is a surprising result considering the high dispersal ability of trout and their ability to colonize new environments using the connections of the hydrographic network (Townsend, 1996). However, there are some hypotheses that can help explain this result. First, the time period analyzed is likely still too short to draw firm conclusions, as species distribution shifts as a result of climate change are difficult to detect in a short time period, and because species distributions can be affected by other anthropogenic disturbances (Isaak & Rieman, 2013). Moreover, some studies have reported the effects of summer droughts on trout habitat preferences (Elliott, 2000; Magoulick & Kobza, 2003), highlighting the importance of climate refugia (e.g., deep pools), in providing resistance or resilience opportunities to trout populations. Moreover, it is necessary to consider the resilience of trout in response to climate change in terms of hydrological variations due to local adaptation phenomena (Kovach et al., 2016), and climate change effects could be masked by the buffering effect of density‐dependent intraspecific dynamics (Ayllón et al., 2019). However, there are evident changes in climate and populations that provide reason for concern, and strong variations in flow rate can overcome these compensatory effects, which lead us to predict a severe decline in trout populations in the near future. It is reasonable to assume that, at present, in the investigated area, the *S. trutta* complex has colonized all the available habitats, since the upper stream reaches in the Tiber River basin seem to be characterized by insufficient flow rates and unsuitable habitat conditions for trout.

Another aspect to be taken into account is the interruption of river continuity: In the upper Tiber basin, there are numerous weirs, some of which are insurmountable by the fish fauna, and thus represent an obstacle to the spread of trout. Fragmentation seems to affect the upper part of the watercourses more than the downstream reaches, resulting in a negative correlation with the distance from the source and the watershed area. This result confirmed the high fragmentation that characterizes the headwater habitats in which trout populations are confined. Furthermore, it should be considered that even decreases in flow rate can represent obstacles to the movement of trout (Isaak & Rieman, 2013). Moreover, there is no evidence for a reduction in the trout distribution range, nor in the trout population densities. These results suggest that, at this stage, anthropogenic stressors have not yet led to local extinction phenomena of the species, and could help to explain the fact that relatively few cases of local climate‐induced extinctions or range contractions have been reported in the literature (Isaak & Rieman, 2013; Kovach et al., 2016). The effects of climate change, in particular, are currently evident in the environment, but not yet in trout populations. This hypothesis is confirmed by the low value observed for extinction probability, which decreased with the current speed, while colonization probability increased with elevation. Nevertheless, the significant progressive decrease of *W*_r_ over the three census periods highlights a progressive worsening of the physiological status of trout over time. These results indicate a state of malaise of the populations, even if cases of local extirpation have not yet been documented.

According to the findings of Daufresne, Lengfellner, and Sommer (2009) and Ayllón et al. (2019), low PSD values and high YOY densities, which indicate a high prevalence of juveniles in populations, could be related to climate change. In particular, as shown by mechanistic simulations predicting trends for the Mediterranean trout populations in northern Spain (Ayllón et al., 2019), climate‐induced changes seem to exert a greater influence on the older specimens, since they have higher energetic costs than juveniles, and this is disadvantageous in the context of poor energy input. As a result, climate change could cause larger trout to experience stressful conditions and have decreased growth performance. Another factor to be taken into consideration to explain this result is the fishing catch, which introduces a strong pressure selectively directed toward larger specimens (Lewin, Arlinghaus, & Mehner, 2006), and is usually one of the main causes of this kind of imbalance in the age structure (Braña, Nicieza, & Toledo, 1992).

However, since the present paper refers to a species complex, in the interpretation of our findings, it is important to take into account that, especially in salmonid populations, introgressive hybridization may lead to a hybrid swarm, and could cause outbreeding depression and the breakdown of local adaptation (Muhlfeld et al., 2009).

In conclusion, climate change and decreasing habitat availability seem to play a crucial role in the distribution and ecology of trout populations in the Tiber River basin, representing additional threats to native Mediterranean trout already endangered by the introduction of *S. trutta* (Caputo et al., 2004; Lorenzoni et al., 2006). However, only long‐term monitoring and further analyses focused on microhabitat analysis will be able to confirm this hypothesis, and to clarify the ecological changes driven by slow processes such as climate change (Elliott, 1994).

Considering the provisions of the Intergovernmental Panel on Climate Change (IPCC), which indicate that in the future we can expect more extended drought periods and less precipitation in the Mediterranean region (IPCC, 2019), the ability of trout populations to compensate for the effects induced by climate change may no longer be sufficient to face large decreases in flow rates and further increases in temperature.

After the genetic detection of residual native trout populations in the Mediterranean area, concrete conservation actions are certainly needed for their protection. The establishment of genetic refuges is often not sufficient to guarantee the conservation of native trout populations (Araguas et al., 2017), and in any case, the protected areas should be delimited on the basis of freshwater biodiversity, covering long stretches of the rivers to be truly effective; otherwise, they are destined to fail (Azevedo‐Santos et al., 2019). In addition, on the basis of our ecological and demographic results, in order to restore the environmental conditions suitable for the presence of the Mediterranean trout, in terms of adequate flow rates, the maintenance of the minimum ecological flows should be ensured through a limitation of water extraction.

## CONFLICT OF INTEREST

The authors declare that they have no conflicts of interest.

## AUTHOR CONTRIBUTION

**Antonella Carosi:** Conceptualization (equal); data curation (equal); investigation (equal); methodology (equal); writing – original draft (equal); writing – review & editing (equal). **Lucia Ghetti:** Funding acquisition (equal); investigation (equal); project administration (equal). **Rosalba Padula:** Data curation (equal); methodology (equal); validation (equal). **Massimo Lorenzoni:** Conceptualization (equal); funding acquisition (equal); project administration (equal); supervision (equal); writing – original draft (equal); writing – review & editing (equal).

## Supporting information

Appendix S1

## Data Availability

The environmental and *Salmo trutta* complex abundance data were archived in the publicly accessible Dryad Digital Repository https://doi.org/10.5061/dryad.p5hqbzkmj.
