# Supplementary material for: Population status and ecology of the Salmo trutta complex in an Italian river basin under multiple anthropogenic pressures
Source: Ecol Evol. 2020 Jun 9;10(14):7320–33. doi: 10.1002/ece3.6457 (PMC7391546; doi:10.1002/ece3.6457)
Supplement: Supplementary file 1 — Appendix S1 [file ECE3-10-7320-s001.docx]

**Appendix S1**

Appendix I Descriptive statistics of environmental parameters and the *S. trutta* complex population and young-of-the-year (YOY) density over the periods 1998-2004, 2005-2011, and 2012-2018.

|  | **1998-2004** | **2005-2011** | **2012-2018** |
| --- | --- | --- | --- |
|  |  | Mean ± SE (standard error) |  |
| Altitude (m a.s.l.) | 334.49 ± 16.91 | | |
| Average current speed (m s^-1^) | 0.42 ± 0.13 | 0.19 ± 0.02 | 0.16 ± 0.01 |
| Biochemical oxygen demand (BOD_5,_ (mg L^-1^) | 1.76 ± 0.41 | 1.55 ± 0.16 | 1.87 ± 0.20 |
| Chemical oxygen demand (COD, (mg L^-1^) | 8.64 ± 1.16 | 7.20 ± 0.37 | 6.40 ± 0.32 |
| Chlorides (Cl^-^, mg L^-1^) | 14.96 ± 1.16 | 18.86 ± 1.56 | 23.57 ± 3.01 |
| Conductivity (μS cm^-1^) | 563.37 ± 26.89 | 560.98 ± 23.57 | 620.39 ± 36.54 |
| Distance from the source (km) | 24.62 ± 3.45 | | |
| Extended biotic index (EBI, units) | 8.00 ± 0.12 | 7.84 ± 0.21 | 7.76 ± 0.14 |
| Flow rate (m^3^ s ^-1^) | 0.95 ± 0.18 | 0.56 ± 0.14 | 0.90 ± 0.23 |
| Fragmentation degree (units) | 3.84 ± 0.57 | | |
| Ammonia (NH_3_, mg L^-1)^ | 0.06 ± 0.01 | 0.10 ± 0.04 | 0.08 ± 0.01 |
| Nitrogen dioxide (NO_2_, mg L^-1^) | 0.02 ± 0.01 | 0.03 ± 0.01 | 0.02 ± 0.01 |
| Nitrate (NO_3_^-^_,_ mg L^-1^) | 1.34 ± 0.21 | 1.51 ± 0.18 | 1.81 ± 0.42 |
| Oxygen (O_2_, mg l^-1^) | 9.29 ± 0.21 | 9.13 ± 0.23 | 9.86 ± 0.26 |
| pH (units) | 8.19 ± 0.03 | 7.98 ± 0.05 | 8.22 ± 0.03 |
| Phosphates (PO_4_, mg L^-1^) | 0.03 ± 0.01 | 0.06 ± 0.01 | 0.12 ± 0.03 |
| Total phosphorous (Ptot, mg L^-1^) | 0.05 ± 0.01 | 0.09 ± 0.02 | 0.12 ± 0.03 |
| *Salmo trutta* complex population density (ind ha ^-1^) | 3190 ± 644 | 2580 ± 427 | 7236 ± 3088 |
| *Salmo trutta* complex young-of-the-year density (ind ha ^-1^) | 1906 ± 441 | 1190 ± 309 | 4761 ± 2693 |
| Sulphate (SO_4_^2-^, mg L^-1^) | 47.97 ± 4.41 | 57.34 ± 7.71 | 61.60 ± 9.51 |
| Water temperature (°C) | 11.34 ± 0.32 | 15.27 ± 0.65 | 14.73 ± 0.50 |
| Watershed area (km^-2^) | 177.68 ± 31.99 | | |

**
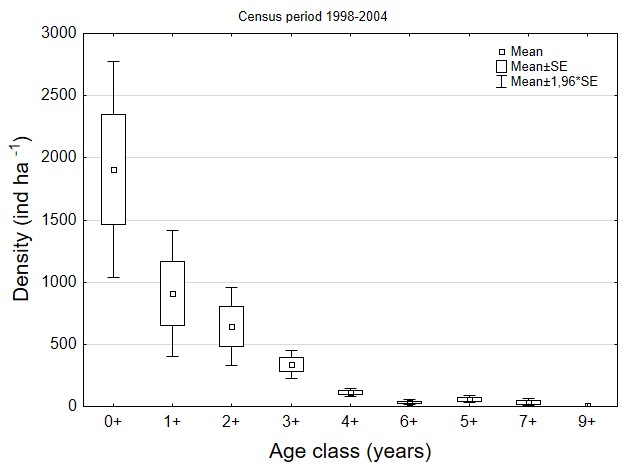

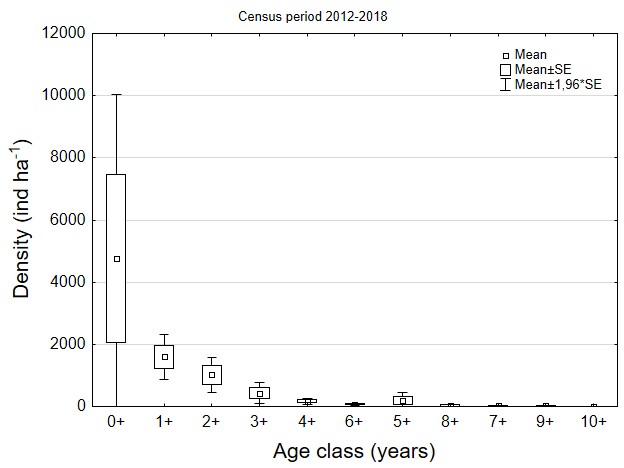

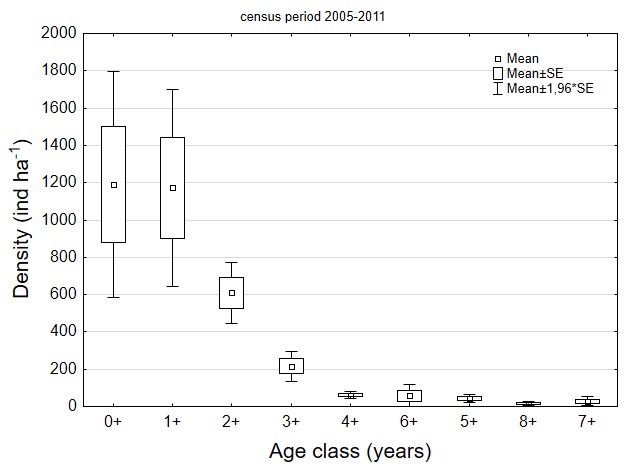

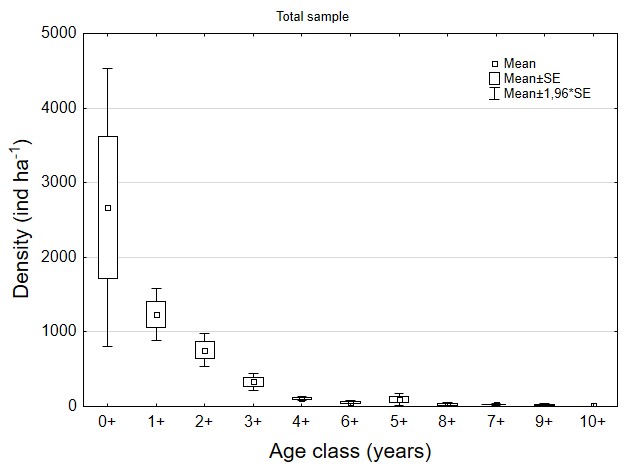
**Appendix II Age structure of the *S. trutta* complex for the total sample (a), and age-based demographic trends over the census periods: b) 1998-2004, c) 2005-2011, d) 2012-2018.

d)

b)

c)

a)
